# Evaluation of the Microba Community Profiler for Taxonomic Profiling of Metagenomic Datasets From the Human Gut Microbiome

**DOI:** 10.3389/fmicb.2021.643682

**Published:** 2021-04-20

**Authors:** Donovan H. Parks, Fabio Rigato, Patricia Vera-Wolf, Lutz Krause, Philip Hugenholtz, Gene W. Tyson, David L. A. Wood

**Affiliations:** ^1^Microba Life Sciences Limited, Brisbane, QLD, Australia; ^2^Australian Centre for Ecogenomics, School of Chemistry and Molecular Biosciences, The University of Queensland, St. Lucia, QLD, Australia; ^3^Centre for Microbiome Research, School of Biomedical Sciences, Translational Research Institute, Queensland University of Technology, Woolloongabba, QLD, Australia

**Keywords:** metagenomics, metagenomic profiling, taxonomic classification, human gut microbiome, benchmarking

## Abstract

A fundamental goal of microbial ecology is to accurately determine the species composition in a given microbial ecosystem. In the context of the human microbiome, this is important for establishing links between microbial species and disease states. Here we benchmark the Microba Community Profiler (MCP) against other metagenomic classifiers using 140 moderate to complex *in silico* microbial communities and a standardized reference genome database. MCP generated accurate relative abundance estimates and made substantially fewer false positive predictions than other classifiers while retaining a high recall rate. We further demonstrated that the accuracy of species classification was substantially increased using the Microba Genome Database, which is more comprehensive than reference datasets used by other classifiers and illustrates the importance of including genomes of uncultured taxa in reference databases. Consequently, MCP classifies appreciably more reads than other classifiers when using their recommended reference databases. These results establish MCP as best-in-class with the ability to produce comprehensive and accurate species profiles of human gastrointestinal samples.

## Introduction

Identifying the microbial species present in natural biological samples is essential for understanding their role in a range of applications including developing diagnostics and therapeutics for human health (Greenblum et al., 2012; Lloyd-Price et al., 2016; Gentile and Weir, 2018; Zmora et al., 2019), refining agricultural practices (Kennedy and Smith, 1995; Orellana et al., 2018), and gaining insights into biogeochemical cycles (Kuypers et al., 2018; Evans et al., 2019). Our inability to culture most *in situ* populations has severely limited our understanding of microbial ecosystems (Epstein, 2013; Lloyd et al., 2018), and it is estimated that even highly studied habitats such as the human gut lack cultured representatives for the majority of species (Almeida et al., 2021). Metagenomics, the sequencing of DNA extracted directly from clinical and environmental ecosystems, has emerged as a powerful approach to bypassing this cultivation bottleneck, providing a holistic view of both the taxonomic and functional diversity of microbial communities (Hugenholtz and Tyson, 2008). This approach has been driven by exponential increases in sequencing throughput and associated decreasing costs leading to the widespread adoption of metagenomics by environmental and clinical researchers.

Metagenomics provides a relatively unbiased sampling of all populations within a community, including bacteria, archaea, eukaryotes, and viruses, and the ability to resolve strains along with genes of interest such as those conferring antimicrobial resistance or pathogenicity (Weinstock, 2012; Köser et al., 2014; Jovel et al., 2016). However, accurately establishing the composition of microbial communities from metagenomic data remains a challenge due to their complexity, the comparatively short read length of the most widely used sequencing technologies (typically 150–250 bp), and incomplete genome reference databases (Sczyrba et al., 2017; Ye et al., 2019). This latter limitation is being addressed by recent approaches that recover high-quality metagenome-assembled genomes (MAGs) from metagenomic datasets resulting in the availability of tens of thousands of draft genomes of uncultured taxa, most notably from the human gastrointestinal tract (Almeida et al., 2019; Nayfach et al., 2019; Pasolli et al., 2019).

Several approaches have been proposed for taxonomically classifying metagenomic data in order to estimate the relative abundance of species in a sample. Metagenomic reads are classified on the basis of sequence similarity to a reference database of previously characterized sequence data, often whole-genome assemblies. Existing metagenomic classifiers can be divided into four groups based on how they establish sequence similarity; namely, (i) genome nucleotide alignment approaches such as Centrifuge (Kim et al., 2016), (ii) protein alignment approaches such as Kaiju (Menzel et al., 2016) and DIAMOND (Buchfink et al., 2015), (iii) marker gene approaches such as MetaPhlAn (Segata et al., 2012) and mOTUs (Milanese et al., 2019), and (iv) composition or k-mer-based approaches such as Kraken (Wood et al., 2019), Bracken (Lu et al., 2017), MetaCache (Müller et al., 2017), and Ganon (Piro et al., 2020). In general, k-mer-based approaches are the most computationally efficient, although have high memory requirements. Marker-based approaches typically have lower memory requirements but at the cost of only classifying reads from a specific subset of genes or genomic regions. Alignment-based approaches favor the additional information provided from mapping reads to reference sequences at the cost of higher computational requirements than k-mer-based approaches and higher memory requirements than marker-based approaches.

The Microba Community Profiler (MCP) was developed to be a highly accurate and specific tool to estimate the relative abundance of bacterial, archaeal, eukaryotic, and viral community members by aligning metagenomic reads to a high-quality and comprehensive database of microbial reference MAGs and isolate genomes. Similar to other classifiers, MCP provides per-read classifications along with an estimate of the proportion of reads assigned to a species. MCP also explicitly indicates the species predicted to be present in a community profile, in contrast to the majority of classifiers considered in this study which report thousands of false positive (FP) species if profiles are not manually inspected and appropriately filtered. The community profiles produced by the MCP are based on the rank normalized taxa and comprehensive species clusters defined by the Genome Taxonomy Database (GTDB; Parks et al., 2018, 2020) which provides improved taxonomic resolution compared to the NCBI Taxonomy (Federhen, 2015; Parks et al., 2020). Here we benchmark MCP against a range of widely used academic metagenomic classifiers using 140 *in silico* mock communities of varying complexity. We demonstrate that MCP has superior recall and precision and maps a higher proportion of reads from gut metagenome datasets than the other classifiers evaluated.

## Results

### Metagenomic Classifiers and Standardized Reference Database

We evaluated the performance of MCP and nine publicly available metagenomic classifiers (Table 1), which use a variety of approaches and have previously been shown to be among the best performing classifiers (Lindgreen et al., 2016; Sczyrba et al., 2017; Ye et al., 2019; Seppey et al., 2020). A single standardized reference database was used by all classifiers in order to evaluate classification performance independent of the reference database (Nasko et al., 2018; Méric et al., 2019; Ye et al., 2019), with the exception of MetaPhlAn2, which was used with its pre-built marker database because building a custom database was not practical. The standardized reference database is comprised of 15,555 quality filtered isolate genomes from 12,250 bacterial and archaeal species (see section “Materials and Methods”; Supplementary Table 1) estimated to have an average completeness and contamination of 99.2 and 0.73%, respectively. Only high-quality isolate genomes were included in the standardized reference database to ensure that classification performance would not be adversely impacted by low genome quality and to reflect that most classifiers recommend the use of reference databases comprised solely of complete isolate genomes (see section “Materials and Methods”). Species were limited to a maximum of five representative genomes in order to reserve a wide diversity of strains for simulating *in silico* mock communities. Species represented by > 1 genome (1,474 of 12,250) had an average intraspecific ANI of 97.8 ± 0.96%. The standardized reference database and comparison of profilers was limited to bacterial and archaeal species as not all classifiers support the classification of eukaryotic or viral species.

**TABLE 1:** Properties of classifiers compared in this study.

Classifier	Version	Classifier type	Base type	References
MCP	2.0.15	Genome	DNA	This study
Ganon	0.1.5	k-mer (k = 19)	DNA	Piro et al., 2020
Kraken	2.0.7	k-mer (k = 35)	DNA	Wood et al., 2019
Bracken	2.5.0	k-mer (k = 35)	DNA	Lu et al., 2017
MetaCache	0.9.0	k-mer (k = 16)	DNA	Müller et al., 2017
Centrifuge	1.0.4	Genome	DNA	Kim et al., 2016
DIAMOND-LCA	0.9.29	Protein	Protein	Buchfink et al., 2015
Kaiju	1.7.2	Protein	protein	Menzel et al., 2016
mOTUs	2.5.1	Marker	DNA	Milanese et al., 2019
MetaPhlAn^#^	2.96.1	Marker	DNA	Truong et al., 2015

*^#^Evaluated using MetaPhlAn database v296 downloaded on February 24, 2020.*

Three parameter settings for the MCP were evaluated: (i) MCP with the standardized reference database and default parameters used to filter out expected FP predictions (referred to as MCP); (ii) MCP without removing expected FPs (referred to as unfiltered MCP or uMCP); and (iii) MCP with default filtering parameters using the Microba Genome Database (MGDB), which comprises 73,646 dereplicated genomes from 28,246 species and is the reference database used by MCP in practice (referred to as MCP-MGDB).

### Simulation of *in silico* Mock Communities

We simulated 140 *in silico* mock microbial communities with varying species diversity, intraspecific diversity, and genomic similarity to reference database genomes (Table 2 and Supplementary Table 2). Communities were comprised of bacterial and archaeal species and simulated with either medium (100 ± 25) or high (500 ± 100) species diversity relative to previously used mocks (Sczyrba et al., 2017), with each species represented by either a single strain or up to 10 randomly selected strains (see section “Materials and Methods”). The average nucleotide identity (ANI) to reference genomes was used to construct mock communities with high (ANI of 99% to 99.75%), moderate (ANI of 97% to 99%), and low (ANI of 95% to 97%) genomic similarity to the standardized reference database. A baseline of 95% ANI was selected to match the commonly used operational definition of a prokaryotic species (Jain et al., 2018; Parks et al., 2020). Mock communities were simulated under all combinations of these parameters, with the exception of mocks with high species diversity and low ANI similarity, as there were insufficient species with available genomes within this lower ANI range. In addition, mock communities were simulated from the reference genomes in order to establish a baseline at 100% ANI similarity for examining the impact of increasing genomic dissimilarity from reference genomes on classifier performance. The 140 mock communities span 6,971 unique species from 2,268 genera and 50 phyla, and contain species ranging from 0.0000019 to 80.5% of the community (Table 2). Communities were simulated to a depth of 2.1 Gb using 2 × 150 bp paired-end reads with strain abundance following a log-normal distribution as this is commonly used for modeling microbial communities (Curtis et al., 2002; Fritz et al., 2019; see section “Materials and Methods”).

**TABLE 2:** Properties of the 140 *in silico* mock communities averaged over the 10 replicates from each class.

ANI similarity	Species diversity	Strain diversity	ANI to closest reference genome (%)	AF to closest reference genome (%)^#^	No.** species	Strains per species	Species abundance (%)
Identical: 100%	Medium	Single	100	100	106 ± 15.8	1	26.8 to 3.3 × 10^–4^
Identical	Medium	Multiple	100	100	92 ± 22.5	2.6 ± 0.16	62.9 to 9.0 × 10^–5^
Identical	High	Single	100	100	490 ± 96.0	1	26.9 to 1.9 × 10^–6^
Identical	High	Multiple	100	100	505 ± 74.8	2.5 ± 0.07	13.2 to 6.1 × 10^–6^
High: [99%, 99.75%]	Medium	Single	99.4 ± 0.22	94.5 ± 3.02	99 ± 21.3	1	38.0 to 2.4 × 10^–4^
High	Medium	Multiple	99.3 ± 0.22	94.4 ± 3.06	106 ± 29.7	4.7 ± 0.33	39.4 to 3.2 × 10^–4^
High	High	Single	99.4 ± 0.22	94.5 ± 2.93	499 ± 86.1	1	60.3 to 1.6 × 10^–5^
High	High	Multiple	99.3 ± 0.22	94.4 ± 3.00	450 ± 116	4.0 ± 0.32	18.4 to 1.3 × 10^–5^
Moderate: [97%, 99%)	Medium	Single	98.3 ± 0.54	90.9 ± 4.41	104 ± 24.3	1	62.3 to 2.3 × 10^–4^
Moderate	Medium	Multiple	98.4 ± 0.52	91.2 ± 3.94	106 ± 19.6	4.7 ± 0.16	29.6 to 3.2 × 10^–4^
Moderate	High	Single	98.3 ± 0.54	90.8 ± 4.23	509 ± 58.6	1	23.2 to 2.8 × 10^–5^
Moderate	High	Multiple	98.3 ± 0.53	91.1 ± 4.19	532 ± 70.9	3.8 ± 0.26	10.0 to 9.2 × 10^–6^
Low: [95%, 97%)	Medium	Single	96.4 ± 0.50	87.9 ± 4.56	93 ± 32.9	1	80.5 to 2.8 × 10^–4^
Low	Medium	Multiple	96.3 ± 0.52	88.0 ± 4.33	109 ± 26.6	3.2 ± 0.23	36.6 to 1.4 × 10^–4^

*^#^AF, alignment fraction, i.e., percentage of orthologous regions shared between two genomes.*

### Establishing Detection Limits of Classifiers

**FIGURE 1:**
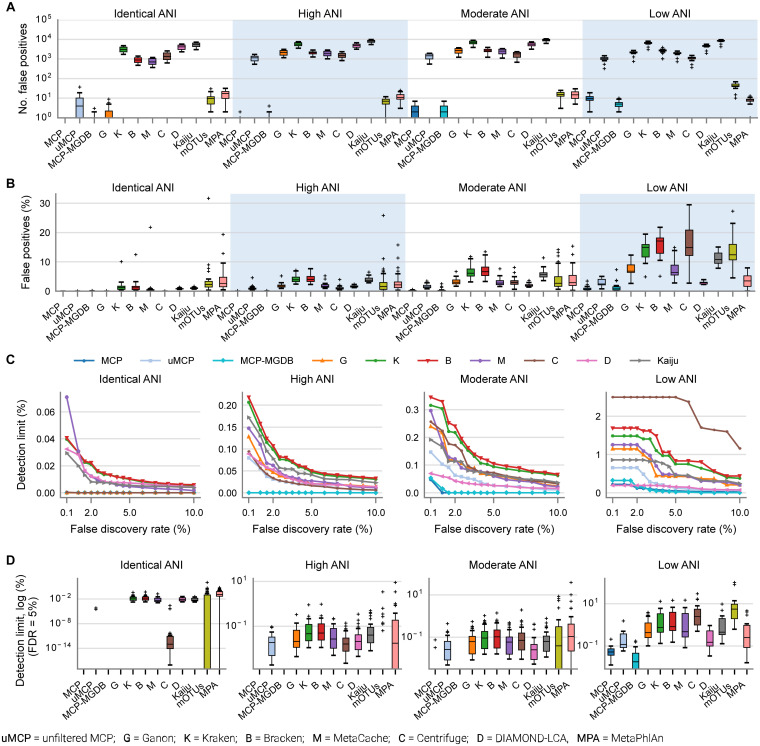
Metagenomic classifiers have different minimum species abundance limits at which species can be reliably detected. **(A)** Number of false positive species predictions made by each classifier on mock communities with decreasing ANI similarity to reference database genomes. **(B)** Percentage of predicted community profiles comprised of false positive predictions. The relatively low community percentages indicate that the majority of false positive predictions are low abundance species. With the exception of the MCP, mOTUs, and MetaPhlAn, these results illustrate that low abundance species must be filtered from the profiles predicted by metagenomic classifiers in order to reduce false positive predictions. **(C)** Median detection limit of each classifier over all mock communities at a given level of ANI similarity to the reference database for varying FDRs. MCP, uMCP, and Ganon report zero false positives for mock communities comprised of genomes in the reference database (identical ANI) and consequently have a median detection limit reported as 0% indicating that all species could be identified without any false positives. Centrifuge and MCP-MGDB only report extremely low abundance false positives for the identical ANI mock communities resulting in median detection limits of 0.00036 and 0%, respectively. Results for mOTUs and MetaPhlAn are not shown as they have substantially higher detection limits than the other classifiers (Supplementary Figure 1 and Table 3). **(D)** Detection limit of each classifier on each mock community resulting in an FDR of 5%. MCP, MCP-MGDB, and Ganon have detection level at or near 0% across all samples at a number of ANI levels so do not produce visible box-and-whisker plots (see Table 3). The box-and-whisker plots show the lower and upper quartiles as a box, the median value as a line within the box, 1.5X the interquartile range as whiskers, and outliers as crosses.

By default, many metagenomic classifiers report all species with any evidence of being present within a sample, down to a single mapped read, which can result in thousands of low abundance FP species predictions, i.e., species not present in the sample (Figures 1A,B; Supplementary Table 3). The implicit expectation is that researchers will filter low abundance predictions or only consider analyses which are insensitive to FP predictions (Ye et al., 2019). Unfortunately, the former is challenging without explicit guidance and the latter is highly restrictive as it limits the ability to confidently assert the presence of low abundance species in a sample. MCP, mOTUs, and MetaPhlAn are exceptions as their predicted community profiles contain only those species with sufficient evidence to assert with high confidence that a species is present in a sample (Figures 1A,B). Consequently, even for the mock communities with high ANI similarity to reference database genomes, the evaluated classifiers report a high proportion of FPs (average of 86.4–96.8% of predicted species), with the exceptions of MCP (0.18 ± 0.44%) and to a lesser extent mOTUs (3.6 ± 2.1%) and MetaPhlAn (7.6 ± 3.7%) (Supplementary Table 3).

Here, the *in silico* mock communities were used to establish detection limits for the different classifiers. Intuitively, the detection limit of a classifier is the lowest abundance species in a sample that can be identified before an unacceptable number of FP species are reported. While the tolerance for FPs is subjective and application-specific, in general FP predictions must be kept low in order to have confidence in the species reported by a classifier. We define the detection limit of each classifier as the lowest reported abundance at which a target false discovery rate (FDR) can be achieved. As expected, the detection limit increases as community members becoming increasingly divergent from genomes in the reference database (Figure 1C). The detection limit also varies substantially between classifiers with MCP having the lowest detection limit regardless of the target FDR (Figure 1C and Table 3). At an FDR of 0.1% (i.e., 1 in 1,000 species expected to be FPs), the MCP had a mean detection limit of 0.0068, 0.069, and 0.52% on mock communities with high, moderate, and low ANI similarity to the reference database, respectively (Table 3). Examining the results at an FDR of 5% illustrates that the detection limit varies substantially for individual mock communities at a specific ANI similarity (Figure 1D). This highlights the challenge in specifying a fixed abundance threshold for filtering classification results which will reliably remove the majority of FP species and, hence, the need for classifiers to directly address the issue of FP predictions.

**TABLE 3:** Mean detection limit of classifiers at select false detection rates.

	High ANI				Moderate ANI				Low ANI			
*Classifier*	*0.1%*	*1%*	*5%*	*10%*	*0.1%*	*1%*	*5%*	*10%*	*0.1%*	*1%*	*5%*	*10%*
MCP	0.0068	0.0016	0	0	0.069	0.048	0.0027	0	0.52	0.52	0.069	0.014
Unfiltered MCP	0.25	0.23	0.023	0.011	0.23	0.21	0.056	0.025	0.98	0.98	0.26	0.079
MCP w/ MGDB	0.014	0.00097	0	0	0.17	0.14	0	0	1.5	1.5	0.037	0.0039
Ganon	0.30	0.27	0.045	0.021	0.38	0.35	0.095	0.046	2.2	2.2	1.1	0.62
Kraken	0.39	0.37	0.097	0.038	0.51	0.47	0.18	0.10	2.6	2.6	2.2	0.9
Bracken	0.41	0.38	0.11	0.042	0.58	0.54	0.21	0.11	2.9	2.9	2.6	1.1
MetaCache	0.36	0.33	0.049	0.021	0.49	0.43	0.098	0.037	2.7	2.7	2.2	0.55
Centrifuge	0.25	0.22	0.026	0.012	0.49	0.45	0.16	0.061	5.5	5.5	5.3	4.6
DIAMOND-LCA	0.14	0.13	0.042	0.018	0.15	0.15	0.053	0.032	0.33	0.33	0.3	0.12
Kaiju	0.27	0.24	0.085	0.032	0.62	0.60	0.12	0.061	1.8	1.8	1.5	0.56
mOTUs	4.0	3.9	0.13	0	2.6	2.6	0.7	0.041	19	19	18	10
MetaPhlAn	2.3	2.3	0.43	0.0094	2.9	2.9	1.6	0.063	2.5	2.5	0.96	0.15

### Predicting the Presence or Absence of Species

In order to assess the accuracy of species predictions for the different classifiers, we conservatively removed low abundance populations at <0.01% as these have a high probability of being reported as FPs by all classifiers other than the MCP (Figure 1C). Removing lower abundance species ensures more accurate results as it acknowledges that species comprising the long tail of microbial communities (Curtis et al., 2002; Fritz et al., 2019) cannot be identified by most metagenomic classifiers without reporting unacceptable numbers of FPs (Figures 1A,B). The mock communities contained an average of 271.4 ± 205.8 and 210.0 ± 141.9 species before and after removal of species at <0.01% abundance, respectively (Supplementary Table 2).

**FIGURE 2:**
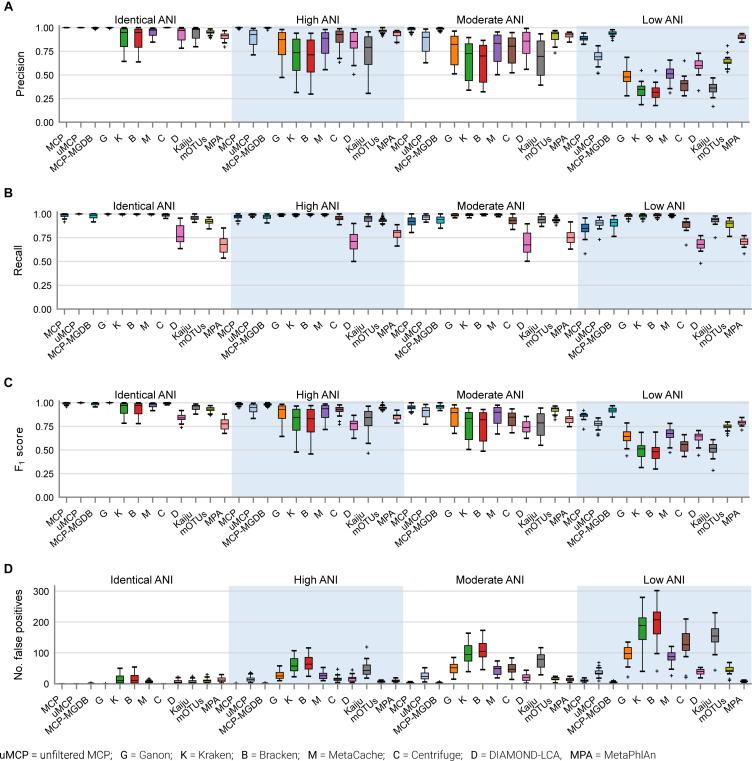
Performance of metagenomic classifiers to predict the presence or absence of species measured using **(A)** precision, **(B)** recall, **(C)** F1 score, and **(D)** number of false positive predictions on mock communities with decreasing ANI similarity to reference database genomes.

The performance of classifiers generally decreased with increasing ANI divergence from the reference database (Figure 2, Table 4, and Supplementary Table 4), consistent with previous studies showing the importance of using a comprehensive reference database (Méric et al., 2019; Piro et al., 2020). MCP reported the lowest number of FP species as indicated by its high precision (Figure 2A). However, there is typically a trade-off between precision and recall, and this is reflected in MCP failing to identify some species whereas other classifiers such as MetaCache and Bracken have high recall with low relative precision (Figures 2A,B). MCP using the MGDB database with equal weight given to precision and recall has the best overall performance (*F*_1_ = 0.97 averaged across all mocks; Figure 2C and Table 4), which demonstrates the positive impact of using a large, comprehensive reference database. Among the classifiers using the standardized reference database, MCP has the best performance across all mock communities (*F*_1_ = 0.96) followed by the uMCP profiles (*F*_1_ = 0.92), mOTUs (*F*_1_ = 0.91), and MetaCache (*F*_1_ = 0.88) (Figure 2C and Table 4). MetaPhlAn performs relatively poorly (*F*_1_ = 0.81) despite using a reference database built from nearly six times as many genomes as the standardized reference database illustrating that a comprehensive database is not sufficient in and of itself to provide good performance.

**TABLE 4:** Evaluation of classifiers to predict the presence or absence of species across the 140 mock communities with and without optimizing the F_1_ score (mean ± std. dev.).

Classifier	Precision	Recall	F_1_ score	Precision (F_1_ optimized)	Recall (F_1_ optimized)	Optimized F_1_ score
MCP	0.98 ± 0.04	0.94 ± 0.06	0.96 ± 0.05	–	–	–
Unfiltered MCP	0.88 ± 0.13	0.97 ± 0.04	0.92 ± 0.09	0.94 ± 0.07	0.95 ± 0.06	0.94 ± 0.06
MCP w/ MGDB	0.99 ± 0.02	0.95 ± 0.04	0.97 ± 0.03	–	–	–
Ganon	0.81 ± 0.20	0.99 ± 0.01	0.87 ± 0.14	0.91 ± 0.10	0.94 ± 0.07	0.92 ± 0.08
Kraken	0.69 ± 0.24	0.99 ± 0.01	0.79 ± 0.18	0.87 ± 0.11	0.91 ± 0.09	0.88 ± 0.09
Bracken	0.68 ± 0.24	1.00 ± 0.01	0.78 ± 0.19	0.87 ± 0.11	0.90 ± 0.10	0.88 ± 0.09
MetaCache	0.80 ± 0.18	0.99 ± 0.01	0.88 ± 0.12	0.90 ± 0.09	0.95 ± 0.05	0.92 ± 0.07
Centrifuge	0.82 ± 0.21	0.95 ± 0.05	0.86 ± 0.15	0.89 ± 0.13	0.90 ± 0.10	0.90 ± 0.11
DIAMOND-LCA	0.83 ± 0.15	0.72 ± 0.11	0.76 ± 0.09	0.87 ± 0.11	0.70 ± 0.10	0.77 ± 0.08
Kaiju	0.73 ± 0.23	0.95 ± 0.04	0.80 ± 0.17	0.87 ± 0.12	0.87 ± 0.09	0.87 ± 0.10
mOTUs	0.90 ± 0.11	0.92 ± 0.03	0.91 ± 0.07	0.91 ± 0.10	0.92 ± 0.04	0.91 ± 0.07
MetaPhlAn	0.92 ± 0.03	0.73 ± 0.08	0.81 ± 0.07	0.92 ± 0.03	0.73 ± 0.08	0.81 ± 0.05

*MCP and MCP w/MGDB were run with default settings without F_1_ optimization.*

Microba Community Profiler provided the best overall performance without the need for manual thresholding because it automatically filters species profiles based on the number of stringently mapped reads being assigned to a species. By contrast, all other classifiers, with the exception of mOTUs and MetaPhlAn, report large numbers of FPs despite limiting results to species at ≥0.01% relative abundance (Figure 2D). In order to further explore the performance of MCP relative to the other classifiers, profiles were filtered at the species abundance resulting in the highest *F*_1_ score as determined independently for each classifier on each mock community (referred to as the optimized *F*_1_ score). Notably, the average MCP *F*_1_ score without optimization of 0.96 is higher than the optimized *F*_1_ score of all other classifiers (Table 4). Establishing the *F*_1_ optimized species abundance threshold is not possible on real data and any fixed abundance threshold will result in the same or worse performance than achieved with these optimized thresholds (Figure 1D).

### Estimating the Relative Abundance of Species

**FIGURE 3:**
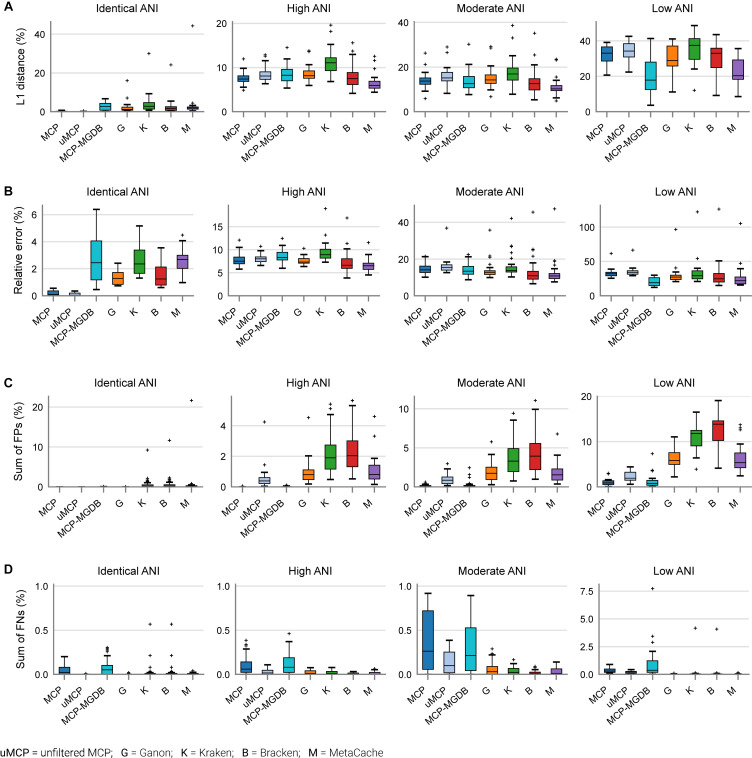
Performance of metagenomic classifiers to predict species abundances. **(A)** L1 distance (0% = identical to ground truth; 200% = no species in common with ground truth) between the ground truth and predicted species profiles. **(B)** Mean relative error of species present in both the ground truth and predicted species profiles. **(C)** Sum of false positive species abundances. **(D)** Sum of false negative species abundances. Lower values indicate better performance. Results are provided across mock communities of increasing ANI divergence from the reference database. Results for Centrifuge, DIAMOND-LCA, Kaiju, and mOTUs are not shown as they have substantially worse species abundance estimates than the other classifiers (Supplementary Figure 2).

Based on mock community analysis (mocks filtered at ≥0.01%), the accuracy of relative abundance estimates decreased with increasing ANI divergence from the reference database (Figure 3, Table 5, and Supplementary Table 5). Centrifuge, DIAMOND-LCA, Kaiju, MetaPhlAn, and to a lesser extent mOTUs deviate substantially from the expected species abundances (Table 5), consistent with prior benchmarking of these classifiers (Ye et al., 2019). The other classifiers have similar overall accuracy in terms of L1 distance (i.e., absolute differences between profiles) with MetaCache (9.0%) performing the best followed by MCP-MGDB (10.0%), MCP (10.8%), and Bracken (11.1%) (Figure 3A and Table 5). Results were similar for the relative absolute percent error with MetaCache having a 1–2% overall improvement over the other classifiers (Figure 3A and Table 5). These results indicate that MCP, MCP-MGDB, Ganon, Kraken, Bracken, and MetaCache are all able to provide reasonably accurate species abundance estimates although the obtained accuracy depends heavily on the similarity of community members to genomes in the reference database. This is seen most clearly with the low ANI similarity mock communities where the abundance estimates are substantially less accurate and more variable (Figure 3A).

**TABLE 5:** Performance statistics for species abundance estimates across the 140 mock communities (mean ± std. dev.).

Classifier	L1 distance	Relative error (%)	Abundance of FPs (%)	Abundance of FNs (%)
MCP	10.8 ± 10.47	11.2 ± 10.97	0.18 ± 0.45	0.20 ± 0.25
Unfiltered MCP	11.3 ± 10.98	11.9 ± 11.92	0.73 ± 0.96	0.08 ± 0.12
MCP w/ MGDB	10.0 ± 7.60	10.1 ± 6.69	0.27 ± 0.82	0.31 ± 0.78
Ganon	11.6 ± 9.90	10.8 ± 11.32	1.72 ± 2.32	0.03 ± 0.05
Kraken	14.4 ± 11.39	12.9 ± 13.53	3.42 ± 3.89	0.06 ± 0.35
Bracken	11.1 ± 10.33	10.5 ± 13.50	3.92 ± 4.53	0.05 ± 0.35
MetaCache	9.0 ± 8.05	10.0 ± 11.46	1.97 ± 3.00	0.02 ± 0.03
Centrifuge	49.0 ± 22.18	52.7 ± 24.92	2.97 ± 5.73	0.28 ± 0.32
DIAMOND-LCA	78.1 ± 9.54	68.6 ± 7.27	0.54 ± 0.53	3.05 ± 2.41
Kaiju	42.5 ± 13.48	34.6 ± 11.20	2.19 ± 2.59	0.20 ± 0.19
mOTUs	18.26 ± 11.86	37.2 ± 34.86	4.79 ± 5.76	4.58 ± 4.69
MetaPhlAn	43.3 ± 19.97	37.9 ± 40.37	3.58 ± 3.21	16.63 ± 10.82

The high precision of the MCP (Figure 2A) resulted in only a small percentage of the predicted community being comprised of FP species (0.18 ± 0.45%; Table 5). This is in contrast to the other classifiers which predict more FP species at an appreciably higher percentage of the community, e.g., MetaCache at 1.97 ± 3.0% and Bracken at 3.92 ± 4.53% (Figure 3C and Table 5). The low ANI similarity mock communities best highlight the tradeoff between the MCP and a more lenient classifier such as MetaCache where FP species account for 1.0 ± 0.77 and 6.6 ± 3.2% of the reported communities, respectively (Figure 3C and Supplementary Table 5). Classifiers generally only fail to identify low abundance species with MCP showing slightly decreased performance as expected from its lower recall rate (Figures 2B, 3D and Table 5). This again highlights the trade-off between false negative (FN) and FP predictions, and illustrates that the MCP favors a slight increase in the percentage of the community that is undetected (Figure 3D) in order to substantially reduce the percentage of the reported community comprised of erroneously identified species (Figure 3C).

### Comparison of Metagenomic Classifiers on Human Gastrointestinal Metagenomes

Community profiles produced by MetaCache, Kraken, Bracken, mOTUs, and MetaPhlAn were compared to those obtained using the MCP on a set of 33 US fecal metagenomes with between 6 and 7 million paired reads from three distinct studies (Supplementary Table 6). These studies were selected in order to evaluate classifiers on fecal samples processed by different labs, from individuals under a range of conditions, and with a read length and sequence depth similar to the *in silico* samples. We focused on these classifiers as they were the strongest performing classifiers on the *in silico* mock communities and/or are widely used by the research community. Unlike the *in silico* mock community analysis, here each classifier was evaluated using its recommended reference database. MCP uses the MGDB which consists of 73,646 dereplicated genomes spanning 28,246 species clusters (see section “Properties of the Microba Genome Database”). MetaCache uses a reference database comprising 16,488 bacterial, 343 archaeal, and 8,999 viral genomes annotated as complete in RefSeq. Kraken and Bracken use a slightly expanded set of 18,871 bacterial, 360 archaeal, and 9,334 viral genomes along with a human reference genome and a collection of known vectors. mOTUs uses a pre-built database of marker genes obtained from ∼25,000 bacterial and archaeal reference genomes which have been supplemented with additional marker genes obtained from public metagenomes. The MetaPhlAn database consists of ∼1.5 million unique clade-specific marker genes obtained from ∼100,000 bacterial, archaeal, and eukaryotic genomes. Species profiles for all classifiers are defined according to the NCBI Taxonomy (Federhen, 2015) with the exception of the MCP which uses the GTDB taxonomy (Parks et al., 2020).

**FIGURE 4:**
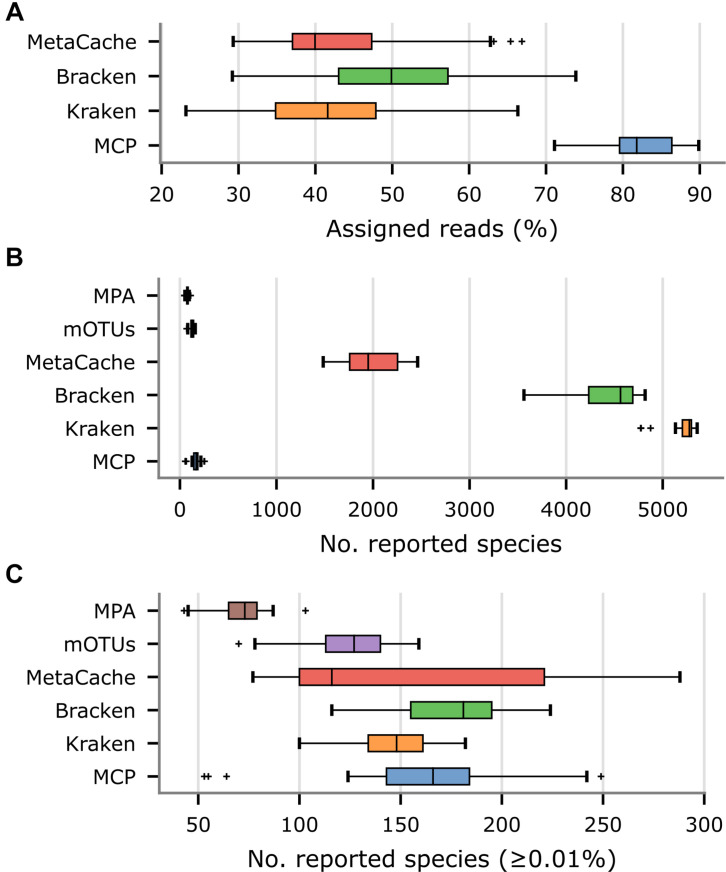
Comparison of metagenomic classifiers on 33 US fecal samples. Community profiles were produced by each classifier using their recommended reference database. **(A)** Percentage of reads assigned to a species in community profiles. **(B)** Number of species reported by each classifier. **(C)** Number of species reported by each classifier with an estimated abundance ≥ 0.01%.

Since the community composition of the fecal samples is unknown, other measurable aspects of the community profiles produced by each metagenomic classifier were evaluated. The percentage of reads assigned to a species was substantially higher for the MCP (82.4% on average) than Kraken (43.3% on average), Bracken (51.3% on average), or MetaCache (43.8% on average; Figure 4A and Supplementary Table 6). This was attributed to the large number of uncultured gut microbiome species represented in MGDB that are absent from the reference databases used by the other classifiers. By design, mOTUs and MetaPhlAn only classify the small subset of reads that map to marker genes and thus assessing percentage of mapped metagenomic reads is not a meaningful comparison. As expected, Kraken, Bracken, and MetaCache report thousands of species (Figure 4B), the great majority of which are likely low abundance FPs based on mock community results (Figures 1A,B). Consequently, species with an estimated abundance <0.01% were removed as these are expected to be predominately FP predictions. Bracken reports the largest number of species with an abundance ≥0.01% (175.3 on average) followed closely by MCP (162.0 on average), MetaCache (151.6 on average), and Kraken (146.3 on average; Figure 4C and Supplementary Table 6). It is notable that mOTUs (123.7 on average) and MetaPhlAn (71.2 on average) report the fewest species in these samples, but were observed to produce far fewer FPs than Kraken, Bracken, and MetaCache on the *in silico* mock communities (Figure 2D). This suggests that these latter classifiers may only be reporting greater numbers of species than mOTUs as a result of increased numbers of FP predictions.

### Properties of the Microba Genome Database

**FIGURE 5:**
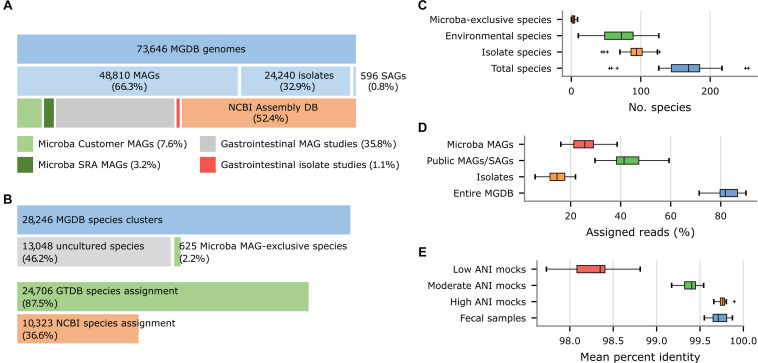
Properties of the Microba Genome Database (MGDB) and the results of profiling 33 US fecal samples using the MCP with the MGDB as a reference database. **(A)** Proportion of MAGs, isolates, and SAGs within the 73,646 genomes comprising the MGDB along with the source of these genome assemblies. Gastrointestinal MAGs were recovered from the studies of Almeida et al. (2019), Nayfach et al. (2019), and Pasolli et al. (2019) and gastrointestinal isolates from the studies of Forster et al. (2019) and Zou et al. (2019). **(B)** Proportion of the 28,246 MGDB species clusters comprised exclusively of uncultured genomes (i.e., MAGs or single-amplified genomes) obtained from multiple sources or solely of MAGs recovered by Microba. This is followed by the proportion of MGDB species clusters that can be assigned a GTDB or NCBI species assignment. **(C)** Total number of species reported by the MCP for each sample and the number of these species which are uncultured species or Microba MAG-exclusive species. **(D)** Total percentage of reads assigned by the MCP to genomes in the MGDB and the percentage assigned to isolates, public MAGs/SAGs, or MAGs obtained by Microba. **(E)** Percent identity of reads mapped by the MCP for 33 US fecal samples and *in silico* mock communities with decreasing ANI similarity to genomes in the standardized reference database.

The MGDB, the default reference database for the MCP, consists of 73,646 dereplicated genomes from 28,246 species clusters as defined by the GTDB (Parks et al., 2018, 2020). The 73,646 genomes in the MGDB were selected in order to provide comprehensive coverage of the genomic diversity within each species and with a specific focus on the human gastrointestinal tract. These genomes were obtained from a variety of sources including the NCBI Assembly database (52.4%), recent large-scale efforts to recover human gastrointestinal MAGs (35.7%; Almeida et al., 2019; Nayfach et al., 2019; Pasolli et al., 2019) or isolates (1.2%; Forster et al., 2019; Zou et al., 2019), and Microba’s own initiatives to obtain MAGs from customer samples (7.6%) and public metagenomes (3.2%; Figure 5A). The 73,646 MGDB genomes are predominantly MAGs (66.3%; Figure 5A) in agreement with a recent estimate that ∼70% of microbial species in the human gastrointestinal tract remain to be cultured (Almeida et al., 2021). These MAGs have an average completeness of 89.5 ± 10.0% and contamination of 1.34 ± 1.48% with ∼60% meeting the completeness and contamination criteria used to define high-quality MAGs (Bowers et al., 2017).

Nearly 50% (13,673) of the 28,246 species in the MGDB are comprised solely of uncultured genomes (i.e., MAGs or single-amplified genomes) with 625 species being comprised exclusively of MAGs obtained by Microba (Figure 5B), which is reflected in their taxonomic assignments. Only 36.6% of the 28,246 species clusters in the MGDB have a species assignment in the NCBI Taxonomy (Figure 5B). For this reason, the MGDB and by extension the MCP uses the GTDB as a taxonomic resource as it provides a substantial improvement in taxonomic resolution with 87.5% of the MGDB species having a GTDB species assignment. Furthermore, adoption of the quantitative criteria used by the GTDB to circumscribe taxa allowed the 625 species exclusive to Microba to be readily identified and given temporary placeholder names with appropriate higher taxonomic ranks as determined by the GTDB-Tk (Chaumeil et al., 2020). The lack of taxonomic resolution in the NCBI Taxonomy extends beyond the rank of species with only 56.8 and 62.5% of MGDB species clusters having an NCBI genus or family assignment, respectively. In contrast, 97.9 and 99.8% of MGDB species clusters have GTDB genus or family assignments, respectively.

### Performance of MCP With the MGDB on Mocks and Gastrointestinal Samples

Microba Community Profiler generally performs better on *in silico* mock communities using the more comprehensive MGDB than the relatively small standardized reference database. In particular, use of the MGDB results in an improvement in correct identification of species comprising the mock communities and in the accuracy of species abundance estimates (Tables 4, 5). The proportion of the community resulting from FP (0.18–0.27%) or FN (0.20–0.31%) predictions increases slightly with the use of the MGDB (Table 5). We attribute this to challenges inherent in robustly distinguishing between highly similar species which are more prevalent in the MGDB (28,246 species) compared to the standardized reference database (12,250 species). Low levels of contamination in MAGs within the MGDB may also contribute to the small increase in FPs.

As the MGDB is comprised of a large number of MAGs and isolates specific to the human gastrointestinal microbiome, we expect the benefits of the MGDB to be more pronounced on samples from this habitat than on the *in silico* mock communities. To illustrate this, we further examined the species profiles produced by the MCP using the MGDB on the 33 US fecal samples. The MCP reports an average of 165.7 ± 44.8 species per sample with 82.4 ± 4.7% of reads being mapped to a species in the MGDB (Figures 5C,D). The 7,950 unique MAGs obtained by Microba account for >10% of the genomes comprising the MGDB (Figure 5A) and capture genomic variation within species not accounted for by publicly-available genomes. This is illustrated by MCP mapping reads to 5,590 ± 1,768 genomes on average across the 33 fecal samples and 1,365 ± 459 of these being to Microba recovered MAGs. Notably, 25.3 ± 5.7% of reads have a best mapping to a MAG obtained by Microba (Figure 5D) and 45.5% of samples contain a Microba MAG which accounts for ≥5% of the mapped reads. This highlights the benefits of using a reference database with strains specific to the habitat being studied.

We assessed the similarity of strains found in the human gastrointestinal tract to genomes comprising the MGDB by considering the percent identity (PI) and percent alignment length (PA) of reads mapped by the MCP. Mapped reads had a PI and PA of 99.72 and 99.99%, respectively, averaged over the 33 fecal samples. Comparing these similarity values to the PI observed for the *in silico* mock communities with known ANI to reference genomes suggests strains found in the human gastrointestinal tract generally have high ANI (i.e., >99%) to MGDB reference genomes (Figure 5E and Supplementary Table 7), indicating that it is a comprehensive database for fecal microbiome profiling.

## Discussion

The MCP was developed to provide accurate metagenomic profiles of fecal microbiomes. Here we evaluated the performance of the MCP relative to nine metagenomic classifiers that are widely used and/or have been shown to be among the best performing classifiers (Lindgreen et al., 2016; Sczyrba et al., 2017; Ye et al., 2019; Seppey et al., 2020). Benchmarking was performed using 140 *in silico* mock communities with decreasing ANI similarity to genomes in a standardized reference database. To the best of our knowledge, this is the first benchmarking study to explicitly investigate the impact of genomic similarity to reference database genomes on classification performance. Our results show that the MCP has the highest combined precision and recall (i.e., *F*_1_ score) among all evaluated classifiers indicating that the optimized trade-off between FP and FN predictions used by MCP provides the most accurate community profiles (Figure 2). The strong performance of the MCP was observed across all mock communities demonstrating that it can reliably identify species even when strains are up to 5% divergent at the nucleotide level from genomes in the reference database. This is in contrast to the other evaluated classifiers which showed a substantial reduction in performance on mock communities with low similarity to genomes in the standardized reference database (Figure 2C). We attribute the strong performance of the MCP to the additional information provided by a reference database of genome assemblies as opposed to k-mers or select marker genes (see section “Materials and Methods”). However, the MCP is typical of genome alignment methods in that it has higher computational requirements than these alternative approaches.

Microba Community Profiler, Kraken, Bracken, and Ganon all provide sound estimates of the relative abundance of microbial species in moderate and high ANI mock communities with MetaCache showing slightly better abundance estimates (Figures 3A,B). An advantage of MCP is a smaller portion of FP predictions (Figure 3C) giving researchers confidence in the predicted community profile. All classifiers failed to provide accurate estimates of the abundance of species on the low ANI mock communities (Figures 3A,B) with the standard reference database. While this limitation warrants further investigation to improve classifier performance, inspection of community profiles of fecal samples produced by the MCP when using the MGDB as a reference database suggests that strains found in the human gastrointestinal tract typically have high ANI similarity to MGDB reference genomes (Figure 5E). This is encouraging as the mock community results suggest that low abundance species (<0.01%) can be identified by the MCP with a low FDR when using a reference database containing closely related strains (Figure 1C and Table 3).

Our benchmarking analysis follows the recommendation that classifiers be evaluated independently of their reference database (Ye et al., 2019) as the specific composition of databases can have a considerable impact on classification performance (Nasko et al., 2018; Méric et al., 2019). This is evident from the higher number of reads from human fecal samples that were classified by MCP compared to MetaCache, Kraken, and Bracken using the default reference databases of each classifier (Figure 4A). We attribute the substantially higher percentage of reads classified by MCP, in part, to the use of a more comprehensive human gut microbiome database (Figure 5A), consistent with previous studies showing the benefit of including human gastrointestinal MAGs (Almeida et al., 2019; Nayfach et al., 2019; Pasolli et al., 2019). As the recovery of MAGs is outpacing our ability to culture new species, it is critical for metagenomic classifiers to make use of this additional source of information, including taxonomic frameworks that accommodate uncultivated species, such as GTDB (Parks et al., 2020; Figure 5B).

The majority of evaluated classifiers provide only a partial solution to the goal of establishing which species are present within a community. This is exemplified by the large number of FPs reported by Ganon, Kraken, Bracken, MetaCache, DIAMOND-LCA, and Kaiju (Figure 5D). Ultimately, these classifiers require researchers to investigate the resulting profiles to establish suitable criteria for establishing which species are likely true positives (TPs; Ye et al., 2019). This is in contrast to MCP, mOTUs, and MetaPhlAn which explicitly aim to produce community profiles comprised solely of TP predictions, without user input.

Microba Community Profiler is under ongoing development and MGDB is constantly updated with genomes of newly identified species. Current efforts are focused on improving the accuracy of species abundance estimates by expanding the genomic diversity of gut species captured by the MGDB and exploring if unclassified reads can be assigned to species without increasing FP predictions. Future improvements to the detection limit of MCP include identifying and removing contamination in reference genomes which can result in low abundance FP predictions. While there are opportunities to continue improving the performance of MCP, the results of this study illustrate that the current version of MCP is the best overall classifier. Community profiling with the MCP is available to the public and scientific community as a service provided by Microba Life Sciences^1^.

## Materials and Methods

### Standardized Reference Database for Classifiers

A reference database of 15,555 genomes from 12,250 species was constructed from RefSeq release 97 (Kitts et al., 2016) obtained from NCBI on November 22, 2019 for use by all metagenomic classifiers (Supplementary Table 1). Only isolate genomes estimated to be >90% complete with <5% contamination by CheckM v1.0.13 (Parks et al., 2015 and where the assembly meets the following criteria were considered for inclusion in the database: (i) <500 contigs, (ii) N50 >20 kb, and (iii) <10,000 undetermined bases. In addition, only genomes with species designations forming a 1-to-1 mapping between the GTDB R04-RS89 (Parks et al., 2018) and NCBI (Federhen, 2015; downloaded November 22, 2019) taxonomies were considered to help ensure reference genomes had correct species assignments. This limited the genomes selected for the reference database to those in GTDB R04-RS89 (based on RefSeq release 89), in order to allow recently submitted genomes to be used for generating *in silico* mock communities. A maximum of five genomes were selected for each species in order of assembly quality as defined by Q = completeness – 5 × contamination – 0.05 × (no. contigs) – 0.00005 × (no. undetermined bases), with an additional 100 added to the assembly quality if it was annotated as complete as determined by consulting the “assembly level” annotation at NCBI. In order to avoid having highly similar genomes in the reference database, a genome was only included if it had an ANI < 99% to all other intraspecific genomes as determined with Mash v2.1.1 (Ondov et al., 2016). The reference database contains 10,776 species with exactly one genome and 1,474 species represented by > 1 genome, and these species have an average intraspecific ANI of 97.8 ± 0.96% as determined with FastANI v1.3 (Jain et al., 2018).

### Generation of *in silico* Mock Communities

*In silico* mock communities were constructed from RefSeq release 97 genomes which passed the same filtering criteria used for the standardized reference database, including the requirement of a 1-to-1 mapping between GTDB and NCBI species assignments (see above). The 67,299 genomes in RefSeq release 97 not covered by GTDB R04-R89 were assigned GTDB classifications using GTDB-Tk v0.3.3 (Chaumeil et al., 2020). Intraspecific ANI values between reference database genomes and potential mock community genomes were calculated with FastANI v1.3. These ANI values were used to generate mock communities comprised of genomes which were increasingly divergent from those in the standardized reference database at ANI intervals of [99, 99.75%], [97, 99%), and [95, 97%) (Table 2). In addition, mock communities comprised of genomes in the reference database (ANI = 100%) were considered as these provide a useful point of comparison.

The number of species in a mock community was modeled on a normal distribution with μ (mean number of species) = 100 and σ (standard deviation in number of species) = 25, or μ = 500 and σ = 100, in order to generate medium and high complexity communities, respectively. Communities were constructed with either a single genome selected from each species, or with 2–10 genomes randomly selected from each species. The relative abundance of genomes comprising mock communities was drawn from a log-normal distribution with a mean of 1 and a standard deviation of 2 as commonly used for modeling microbial communities (Curtis et al., 2002; Fritz et al., 2019).

The number of paired reads generated for each genome was *n*_*i*_ = *N*×(*a*_*i*_*s*_*i*_/∑_*j*_*a*_*j*_*s*_*j*_), where *s*_*i*_ is the size of genome *i*, *a*_*i*_ is the relative abundance of genome *i*, and *N* is the total number of paired reads comprising the *in silico* community. All *in silico* communities were simulated to a depth of 2.1 Gb by randomly sampling 2 × 150 bp paired-end reads with an insert size of 200 ± 25 bp across each genome in the mock community.

### Building Custom Databases for Metagenomic Classifiers

The genomes comprising the standardized reference database were used to build a custom database for each classifier using recommended default parameters. Genomes comprising the standardized reference database were contained in individual FASTA files in a single directory (db_genomes) and concatenated into a single FASTA file (db_genomes_all.fna) in order to facilitate the requirements of the different metagenomic classifiers. The custom databases were built using the same NCBI Taxonomy data files used while constructing the standardized reference database which were obtained from NCBI^2^ on November 22 2019 and consist of the files nodes.dmp, names.dmp, merged.dmp, nucl_gb.accession2taxid, and nucl_wgs accession2taxid. DIAMOND and Kaiju require protein sequences which were called for each reference genomes using Prodigal v2.6.3 (Hyatt et al., 2010) and the translation table specified at NCBI: prodigal -c -m -q -f gff -p single -g <trans_table> -i <ref_genome> -a <aa_output>. Prodigal was used to predict protein sequences as NCBI does not provide protein sequences for all genomes comprising the standardized reference database. A mapping file indicating the NCBI species ID for each predicted protein (db_proteins_all.taxid_map.tsv) and a FASTA file containing all proteins (db_proteins_all.faa) were created to facilitate building the DIAMOND and Kaiju databases. The commands executed to build custom databases for each classifier are given in Supplementary Table 8.

### Species-Level Community Profiling With Metagenomic Classifiers

Community profiles were generated for mock communities using each of the metagenomic classifiers run with default parameters (Supplementary Table 9). DIAMOND indicates the lowest common ancestor (LCA) for each query read, but does not produce a profile indicating the proportion of reads assigned to each species. A custom script was used to tabulate the proportion of reads assigned to each species. Reads with an LCA above the rank of species were considered unclassified for the purposes of creating a species profile for each mock community.

MetaPhlAn results were obtained using the v296_CHOCOPhlAn_201901 marker set which may have species assignments that differ from those defined for the *in silico* mock communities due to reclassifications at NCBI. To account for this, the NCBI TaxIds produced by MetaPhlAn were used to establish species names as defined in the November 22, 2019 NCBI Taxonomy data files, the same files used to construct the mock communities.

### Microba Genome Database

The MGDB v2 was built from genomes in GTDB R04-RS89, MAGs obtained from Australian fecal samples, MAGs mined from SRA samples by Microba, and MAGs and isolate genomes from Almeida et al. (2019), Forster et al. (2019), Nayfach et al. (2019), Pasolli et al. (2019), and Zou et al. (2019). Together these sources span 411,415 genomes after removing lower quality assemblies as defined by having a completeness estimate <80%, a contamination estimate >5%, being comprised of >1,000 contigs, or having an N50 < 5 kb. These genomes were dereplicated based on ANI similarity to obtain a final database consisting of 73,646 genomes from 28,246 species. Completeness and contamination estimates for genomes within the MGDB were determined using CheckM v1.1.2 (Parks et al., 2015). Genomes without taxonomic assignments in GTDB R04-RS89 were assigned a GTDB classification using GTDB-Tk v0.3.3 (Chaumeil et al., 2020) and additional species clusters defined using the ANI criteria used by the GTDB (Parks et al., 2020).

### MCP Database Indexing, Read Mapping, and Community Profiling

Microba Community Profiler is propriety software available by contacting Microba Life Sciences at https://microba.com/microbiome-research. MCP is a whole-genome alignment tool, which uses a combination of BWA v0.7.17 (Li and Durbin, 2009), SAMtools v1.7 (Li et al., 2009), and custom software optimized to ensure that reads are assigned to reference genomes that are closely related to the strains comprising a metagenomic sample. Mapping the 140 *in silico* mock communities (each 2.1 Gb, 2 × 150 bp paired-end reads) required 37.6 ± 9.9 min on average and profiling required 14.5 ± 2.4 min on average when using 64 Intel Xeon 2.00 GHz processors.

### Classifier Performance Metrics

Precision and recall can be defined in terms of the number of species correctly (TP) and incorrectly (FP) identified by a classifier along with the number of unidentified species present in a sample (FN). Precision, P = TP/(TP+FP), is the fraction of species identified by a classifier that are correct, while recall, R = TP/(TP+FN), is the fraction of correctly identified species within a sample. The *F*_1_ score is the harmonic mean of precision and recall, (2 × P × R)/(P+R), which weights these terms equally in a single metric.

Absolute and relative percent error for each species within a sample is defined in terms of the true, T, and estimated, E, abundance of a species. Absolute error, A = |T-E|, indicates how close abundances estimates are to the true abundance of a species, while relative percent error, R = 100 × A/T, expresses how large the absolute error is compared to the true abundance which highlights poor estimates of low abundance species. The L1 (Manhattan) distance is the sum of absolute errors across all ground truth and predicted species which provides a measure that incorporates FP predictions (Ye et al., 2019). The mean relative percent error across all ground truth species in a sample was used for assessing classifier performance. Different ground truth abundances were used for classifiers that estimate (i) the relative proportion of reads from each species (Ganon, Kraken, Bracken, MetaCache, DIAMOND-LCA, and Kaiju) and (ii) the relative proportion of reads normalized by genome size (MCP, Centrifuge, mOTUs, and MetaPhlAn).

Previous benchmarking studies have suggested the use of the Euclidean distance and the area under the precision–recall curve (AUPR) for evaluating classifier performance (Ye et al., 2019). We elected to use the L1 distance as it does not give additional weight to high abundance species and report precision and recall independently as the AUPR is known to be biased toward low-precision, high-recall classifiers (Ye et al., 2019). This is a notable limitation as many classifiers fall into this categorization.

### Establishing Classifier Detection Limits

The detection limit for classifiers was defined as the lowest abundance species in a sample that achieved a specified FDRs, FDR = FP/(TP+FP). This was determined by ordering identified species in ascending order of abundance and calculating the FDR after filtering species below each abundance level. The detection limit for a sample is the lowest abundance at which the desired FDR could be achieved.

### Community Profiles for Human Gastrointestinal Metagenomes

Metagenomic data from three published studies of US fecal (Supplementary Table 6) were processed by selected metagenomic classifiers using recommended reference databases. Samples with 2 × 150 bp paired read were considered in order to allow a direct comparison with results obtained on the *in silico* samples. Samples were downloaded from the NCBI Sequence Read Archive (Leinonen et al., 2011) and processed to remove potential human contamination by mapping reads to the human reference genome (GRCh38.p12) using the MEM method of BWA v0.7.17-r1188 (Li and Durbin, 2009). Reads mapped as proper pairs where either read had a PI ≥95% and PA ≥90% were considered human and removed from the sample. Remaining reads were processed using Trimmomatic v0.36 (Bolger et al., 2014) to remove adapters, filter leading or trailing bases with a quality score <3, clip reads when the average 4-base window had a quality score <15, and discard reads <100 bp in length after applying the previous QC steps. Samples with <6 million reads after QC were discarded and samples with >7 million pairs were subsampled to 7 million paired reads using seqtk v1.2-r94^3^ in order to minimize the effect of sequencing depth and make these samples comparable in depth to the *in silico* samples.

Reference databases for MetaCache and Kraken were obtained using the scripts and recommended parameters suggested by these classifiers (Supplementary Table 10). These databases were built on March 3, 2020. Kraken v2.0.8 was used for this analysis as opposed to v2.0.7 as changes to NCBI data formats required the use of this later version. MetaCache and Kraken differ in the set of included reference genomes as MetaCache only considered genomes annotated as a “Complete Genome” at NCBI, while Kraken also includes genomes annotated as “Chromosome.” Bracken results are derived from the mapping information produced by Kraken. mOTUs and MetaPhlAn results were obtained using pre-built marker databases. Profiling was performed as previous described (Supplementary Table 9).

## Data Availability Statement

The *in silico* paired-end reads and ground truth data for the 140 mock communities are available on Zenodo (https://doi.org/10.5281/zenodo.4470159). Genomes used to build the standardized reference database are given in Supplementary Table 1 and can be obtained from the NCBI Assembly database (Kitts et al., 2016). The 33 US fecal samples are available from the NCBI Sequence Read Archive (Supplementary Table 6). The metagenomic classifiers can be obtained from their respective websites as indicated in the cited literature with the exception of MCP which is proprietary software developed by Microba Life Sciences Limited.

## Author Contributions

DP and DW developed the benchmarking framework with recommendations provided by all others authors. FR developed the computational infrastructure used to carry out this study. PV-W identified and performed initial bioinformatic analyses of the US fecal samples. LK reviewed and provided suggestions on early drafts of the manuscript. DP, DW, GT, and PH wrote the manuscript with constructive suggestions from all other authors. All authors contributed to the article and approved the submitted version.

## Conflict of Interest

DP, FR, PV-W, LK, and DW are the employees of Microba Life Sciences. GT and PH are the founders of Microba Life Sciences. Microba Life Sciences is a microbial genomics company developing microbiome-based diagnostics and therapeutics and offers metagenomic gut microbiome reports. The Microba Community Profiler (MCP) is proprietary software of Microba Life Sciences, but is available commercially for researchers in order to facilitate replicating the results of this study.

## Footnotes

1 https://microba.com/microbiome-research/

2 https://ftp.ncbi.nih.gov/pub/taxonomy/

3 https://github.com/lh3/seqtk
