# Supplementary material for: Evaluation of the Microba Community Profiler for Taxonomic Profiling of Metagenomic Datasets From the Human Gut Microbiome
Source: Front Microbiol. 2021 Apr 20;12:643682. doi: 10.3389/fmicb.2021.643682 (PMC8093879; doi:10.3389/fmicb.2021.643682)
Supplement: Supplementary file 1 [file Presentation_1.zip › Supplementary Materials/Supplementary Material.pdf]

Supplementary Material

1 Supplementary Figures and Tables

1.1 Supplementary Figures

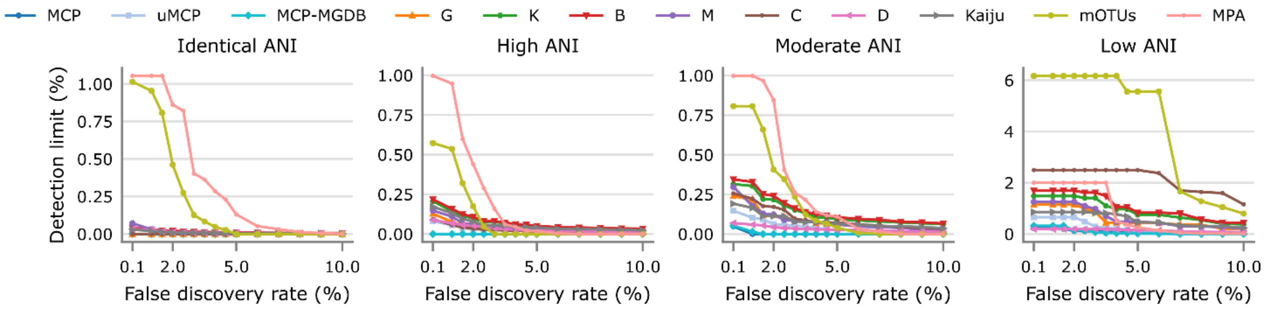

Supplementary Figure 1. Median detection limit of each classifier, including mOTUs and MPA, over all mock communities at a given level of ANI similarity to the reference database for varying false discovery rates. uMCP = unfiltered MCP; G = Ganon; K = Kraken; B = Bracken; M = MetaCache; C = Centrifuge; D = DIAMOND-LCA, MPA = MetaPhlAn.

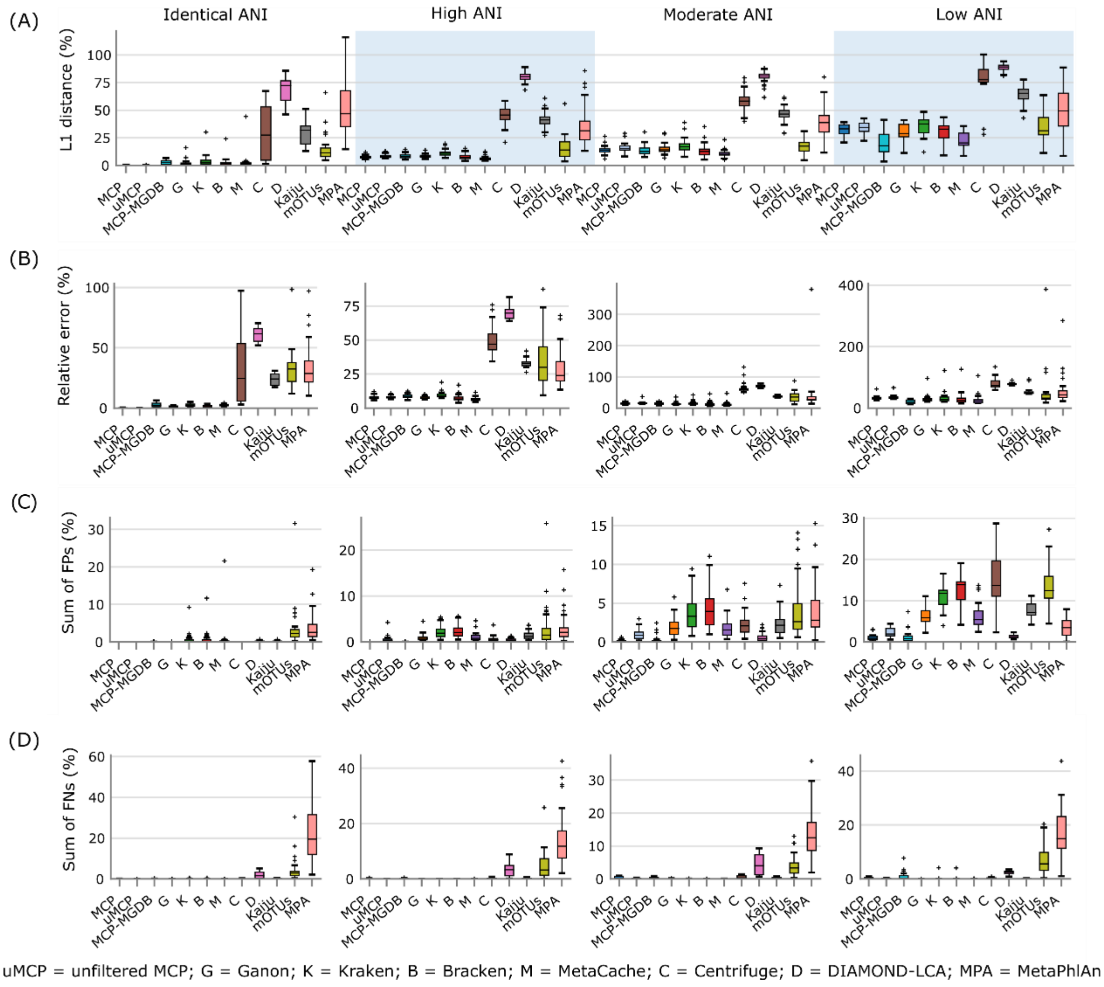

Supplementary Figure 2. Performance of metagenomic classifiers to predict species abundances. (A) L1 distance (0% = identical to ground truth; 200% = no species in common with ground truth) between the ground truth and predicted species profiles. (B) Mean relative error of species present in both the ground truth and predicted species profiles. (C) Sum of false positive species abundances. (D) Sum of false negative species abundances. Identical to Figure 3 except results for Centrifuge, DIAMOND-LCA, Kaiju, mOTUs, and MetaPhlAn are included.

1.2 Supplementary Tables

Supplementary Table 1. Metadata for the 15,555 isolate genomes comprising the standardized reference database (see Excel file).

Supplementary Table 2. Metadata for the 140 *in silico* mock communities (see Excel file).

Supplementary Table 3. Number of true positive (TP) and false positive (FP) species predictions along with the false discovery rate (FDR) of metagenomic classifiers on mock communities without filtering of low abundance species and with varying ANI to reference database genomes (mean ± std. dev.).

	High ANI			***Moderate ANI***			***Low ANI***		
Classifier	*TP*	*FP*	*FDR*	*TP*	*FP*	*FDR*	*TP*	*FP*	*FDR*
MCP	214 ± 129.6	0.20 ± 0.46	0.18 ± 0.44	217 ± 130.1	2.52 ± 1.83	1.75 ± 1.89	75 ± 21.88	9.90 ± 4.45	11.34 ± 3.40
Unfiltered MCP	288 ± 201.0	1105 ± 329.5	81.7 ± 7.88	312 ± 213.2	1407 ± 425.2	84.0 ± 6.77	101 ± 30.8	1049 ± 263.2	91.2 ± 1.66
MCP w/ MGDB	213 ± 128.4	0.47 ± 0.77	0.27 ± 0.50	222 ± 133.7	2.42 ± 1.87	1.54 ± 1.40	81 ± 23.98	5.10 ± 2.23	6.14 ± 2.66
Ganon	288 ± 201.1	2148 ± 599.3	89.6 ± 4.81	312 ± 213.2	2673 ± 754.7	90.8 ± 4.19	101 ± 30.8	2154 ± 439.2	95.6 ± 0.84
Kraken	288 ± 201.1	5756 ± 1188.2	95.0 ± 2.36	312 ± 213.2	7003 ± 1338.6	96.1 ± 2.12	100 ± 30.8	6470 ± 1128.8	98.5 ± 0.33
Bracken	287 ± 200.1	2057 ± 367.0	88.7 ± 6.34	312 ± 212.6	2677 ± 559.4	90.6 ± 5.10	100 ± 30.8	2618 ± 638.5	96.2 ± 1.18
MetaCache	288 ± 201.0	1968 ± 533.8	88.7 ± 5.27	312 ± 213.2	2457 ± 676.9	90.1 ± 4.57	101 ± 30.8	1942 ± 406.6	95.1 ± 0.96
Centrifuge	288 ± 201.0	1584 ± 451.7	86.4 ± 6.26	312 ± 212.7	1716 ± 500.9	86.4 ± 5.97	100 ± 30.6	1097 ± 279.7	91.6 ± 1.77
DIAMOND-LCA	288 ± 200.9	4828 ± 953.3	94.9 ± 2.82	312 ± 213.0	5617 ± 1103.3	95.3 ± 2.58	101 ± 30.9	4611 ± 868.0	97.9 ± 0.48
Kaiju	288 ± 201.1	7943 ± 1177.9	96.8 ± 1.92	312 ± 213.2	9161 ± 1094.7	96.9 ± 1.85	101 ± 30.8	8385 ± 1247.4	98.8 ± 0.27
mOTUs	218 ± 136.0	6.67 ± 2.94	3.64 ± 2.05	240 ± 151.5	15.9 ± 5.30	8.58 ± 5.47	82 ± 25.3	44.65 ± 14.37	35.1 ± 6.00
MetaPhlAn	187 ± 113.6	12.2 ± 5.25	7.55 ± 3.72	193 ± 117.5	15.5 ± 6.58	8.72 ± 2.92	65 ± 19.6	7.85 ± 2.50	10.7 ± 2.63

Supplementary Table 4. Evaluation of classifiers to predict the presence or absence of species in mock communities with varying ANI to reference database genomes (mean ± std. dev.).

	High ANI			***Moderate ANI***			***Low ANI***		
Classifier	*Precision*	*Recall*	*F_1 score*	*Precision*	*Recall*	*F_1 score*	*Precision*	*Recall*	*F_1 score*
MCP	1.00 ± 0.00	0.96 ± 0.03	0.98 ± 0.01	0.98 ± 0.02	0.92 ± 0.05	0.95 ± 0.02	0.89 ± 0.03	0.85 ± 0.07	0.86 ± 0.03
Unfiltered MCP	0.90 ± 0.09	0.99 ± 0.01	0.98 ± 0.01	0.84 ± 0.12	0.96 ± 0.03	0.89 ± 0.06	0.68 ± 0.09	0.90 ± 0.05	0.77 ± 0.05
MCP w/ MGDB	1.00 ± 0.00	0.97 ± 0.02	0.94 ± 0.05	0.99 ± 0.01	0.93 ± 0.04	0.96 ± 0.02	0.94 ± 0.03	0.90 ± 0.06	0.92 ± 0.03
Ganon	0.83 ± 0.13	0.99 ± 0.01	0.98 ± 0.01	0.76 ± 0.16	0.99 ± 0.01	0.85 ± 0.10	0.48 ± 0.09	0.98 ± 0.02	0.64 ± 0.08
Kraken	0.71 ± 0.18	0.99 ± 0.01	0.90 ± 0.08	0.64 ± 0.20	0.99 ± 0.01	0.76 ± 0.15	0.34 ± 0.08	0.98 ± 0.02	0.50 ± 0.09
Bracken	0.70 ± 0.18	1.00 ± 0.00	0.82 ± 0.13	0.62 ± 0.20	0.99 ± 0.01	0.74 ± 0.16	0.32 ± 0.08	0.99 ± 0.01	0.48 ± 0.09
MetaCache	0.83 ± 0.13	1.00 ± 0.01	0.81 ± 0.14	0.78 ± 0.15	0.99 ± 0.01	0.86 ± 0.09	0.50 ± 0.08	0.99 ± 0.01	0.66 ± 0.07
Centrifuge	0.89 ± 0.09	0.96 ± 0.03	0.90 ± 0.08	0.76 ± 0.14	0.93 ± 0.04	0.83 ± 0.07	0.40 ± 0.08	0.88 ± 0.06	0.54 ± 0.06
DIAMOND-LCA	0.85 ± 0.12	0.70 ± 0.11	0.92 ± 0.04	0.83 ± 0.13	0.70 ± 0.12	0.74 ± 0.06	0.60 ± 0.09	0.68 ± 0.07	0.63 ± 0.06
Kaiju	0.75 ± 0.17	0.95 ± 0.04	0.76 ± 0.06	0.68 ± 0.19	0.94 ± 0.04	0.77 ± 0.12	0.35 ± 0.07	0.93 ± 0.05	0.51 ± 0.07
mOTUs	0.96 ± 0.02	0.93 ± 0.02	0.82 ± 0.11	0.91 ± 0.05	0.94 ± 0.02	0.92 ± 0.03	0.64 ± 0.06	0.89 ± 0.05	0.75 ± 0.04
MetaPhlAn	0.93 ± 0.03	0.79 ± 0.05	0.95 ± 0.02	0.92 ± 0.03	0.75 ± 0.07	0.82 ± 0.04	0.90 ± 0.03	0.70 ± 0.05	0.79 ± 0.03

Supplementary Table 5. Performance statistics for species abundance estimates on mock communities with varying ANI to reference database genomes (mean ± std. dev.).

	High ANI			*Moderate ANI*			*Low ANI*		
Classifier	*L1 distance*	*Relative error*	*Sum of FPs*	*L1 distance*	*Relative error*	*Sum of FPs*	*L1 distance*	*Relative error*	*Sum of FPs*
MCP	7.63 ± 1.43	7.84 ± 1.32	0.01 ± 0.02	14.2 ± 3.24	14.6 ± 2.60	0.12 ± 0.13	31.9 ± 5.31	33.2 ± 7.36	1.01 ± 0.77
Unfiltered MCP	8.41 ± 1.49	8.04 ± 0.85	0.53 ± 0.66	15.9 ± 3.64	15.8 ± 3.74	0.94 ± 0.63	34.5 ± 5.48	35.6 ± 7.73	2.31 ± 1.14
MCP w/ MGDB	8.39 ± 1.88	8.59 ± 1.35	0.02 ± 0.03	13.6 ± 4.17	14.1 ± 3.10	0.22 ± 0.47	20.2 ± 10.58	20.2 ± 5.98	1.35 ± 1.69
Ganon	8.53 ± 1.66	7.56 ± 0.80	0.93 ± 0.74	15.1 ± 4.17	13.7 ± 4.26	1.90 ± 1.14	30.3 ± 7.36	30.5 ± 15.89	6.38 ± 2.27
Kraken	11.03 ± 2.60	9.41 ± 1.99	2.05 ± 1.20	17.7 ± 5.48	15.6 ± 5.70	3.70 ± 2.08	35.6 ± 8.81	35.1 ± 21.44	11.2 ± 3.28
Bracken	7.66 ± 2.38	7.09 ± 2.31	2.27 ± 1.30	13.2 ± 5.20	12.6 ± 6.86	4.24 ± 2.38	31.0 ± 8.47	31.2 ± 23.43	12.9 ± 3.87
MetaCache	6.41 ± 1.65	6.48 ± 1.32	1.04 ± 0.84	11.0 ± 3.43	12.0 ± 6.27	1.81 ± 1.21	22.6 ± 7.35	27.7 ± 19.52	6.57 ± 3.24
Centrifuge	45.8 ± 7.40	49.0 ± 9.32	0.59 ± 0.60	57.6 ± 8.20	63.1 ± 14.50	2.32 ± 1.41	77.3 ± 17.34	80.7 ± 17.49	15.0 ± 7.20
DIAMOND-LCA	80.3 ± 4.11	69.8 ± 4.15	0.47 ± 0.31	80.5 ± 4.85	70.2 ± 3.53	0.60 ± 0.51	88.4 ± 3.52	78.7 ± 3.56	1.35 ± 0.46
Kaiju	41.6 ± 6.44	33.1 ± 3.11	1.42 ± 0.84	46.5 ± 6.35	37.2 ± 2.29	2.34 ± 1.46	63.4 ± 8.20	54.2 ± 13.01	7.51 ± 1.85
mOTUs	15.1 ± 9.58	33.3 ± 17.48	2.74 ± 4.39	17.3 ± 6.22	35.1 ± 15.14	4.07 ± 3.54	35.0 ± 12.41	59.4 ± 80.10	13.5 ± 5.49
MetaPhlAn	34.6 ± 16.25	28.9 ± 13.52	2.91 ± 3.03	40.0 ± 13.21	39.1 ± 55.33	3.98 ± 3.19	49.5 ± 19.46	63.1 ± 58.48	3.53 ± 2.47

Supplementary Table 6. Profiling results on 33 American fecal samples (see Excel file).

Supplementary Table 7. Mean percent identity (PI) and percent alignment length (PA) of reads mapped to reference databases by the MCP

Samples	*No. samples*	*ANI to reference genomes*	*PI*	*PA*
American fecal samples	33	(unknown)	99.72	99.99
High ANI mock samples	40	99 to 99.75%	99.76	99.98
Moderate ANI mock samples	40	97 to 99%	99.39	99.97
Low ANI mock samples	20	95 to 97%	98.26	99.91

Supplementary Table 8. Building custom databases for each of the metagenomic classifiers.

Classifier	*Command(s)*
Kraken	NCBI Taxonomy data files were placed in kraken2_db/taxonomy and the database built with:<br>> kraken2-build --threads 4 --add-to-library db_genomes_all.fna --db kraken2_db<br>> kraken2-build --threads 64 --build --db kraken2_db
Bracken	Built from Kraken 2 database using:<br>> bracken-build -d kraken2_db -t 60 -k 35 -l 150
Centrifuge	> centrifuge-build -p 96 --conversion-table nucl_wgs_gb.accession2taxid --name-table names.dmp --taxonomy-tree nodes.dmp db_genomes_all.fna centrifuge_db
Ganon	> ganon build -d ganon_db --input-files db_genomes_all.fna --taxdump-file nodes.dmp names.dmp merged.dmp -t 48
DIAMOND-LCA	> diamond makedb -p 40 --db db_proteins_all.faa --in db_proteins_all.faa --taxonmap db_proteins_all.taxid_map.tsv --taxonnodes nodes.dmp
Kaiju	Sequence headers in db_proteins_all.faa were formatted to contain NCBI TaxIds and the database built with:<br>> kaiju-mkbwt -n 20 -o kaiju_db db_proteins_all.faa<br>> kaiju-mkfmi kaiju_db
MetaCache	NCBI Taxonomy data files were placed in the directory ncbi_taxonomy and the database built with:<br>> metacache build metacache_db db_genomes -taxonomy ncbi_taxonomy
mOTUs	A file, genomes.list, containing the accession of all standardized database genomes was created along with a mOTUs 2 formatted taxonomy file, taxonomy_file.txt. Since mOTUs 2 only supports extending an existing database, empty mOTU 2 data files were created in the directory clean_db. The database was then built with:<br>> parallel -j 64 -a genomes.list "extend_mOTUs_addGenome.sh db_genomes/{}.fasta {} standard_db extend_mOTUs_DB/SCRIPTS/ clean_db"<br>> extend_mOTUs_generateDB.sh genomes.list STANDARD_DB taxonomy_file.txt standard_db extend_mOTUs_DB/SCRIPTS/ clean_db
MetaPhlAn	MetaPhlAn 2 was run using the marker dataset v296_CHOCOPhlAn_201901 downloaded on Feb. 25, 2020.

Supplementary Table 9. Profiling mock communities with each of the metagenomic classifiers.

Classifier	*Command(s)*
Kraken	> kraken2 --db {kraken2_db} --report {sample_id}.kreport2 --output {sample_id} --paired {left_reads} {right_reads}
Bracken	> est_abundance.py -i {sample_id}.kreport2 -k {bracken2_db} -o {sample_id}.bracken
Centrifuge	> centrifuge -x {centrifuge_db} -1 {left_reads} -2 {right_reads} --report-file {profile_file} -S {per_read_file}
Ganon	> ganon classify -d {ganon_db} -p {left_reads} {right_reads} -o {profile_file}
DIAMOND-LCA	> diamond blastx --query-gencode 11 -f 102 --top 10 --min-score 50 -d {diamond_db} -q {left_reads} -o {profile_file}
Kaiju	> kaiju -v -t {ncbi_nodes} -f {kaiju_db} -i {left_reads} -j {right_reads} -o {report}<br>> kaiju2table -v -t {ncbi_nodes} -n {ncbi_names} -r species -o { profile_file} {report}
MetaCache	> metacache query {metacache_db} {left_reads} {right_reads} -pairfiles -out {report} -abundances {profile_file} -abundance-per species
mOTUs	> motus profile -f {left_reads} -r {right_reads} -db {motu_db} -o {profile_file}
MetaPhlAn	> metaphlan2.py {left_reads},{right_reads} {profile_file} --bowtie2out {sample_id}.bowtie2.bz2 --index v296_CHOCOPhlAn_201901 --ignore_eukaryotes --input_type fastq

Supplementary Table 10. Commands for building recommended reference databases.

Classifier	*Command(s)*
Kraken	> kraken2-build --standard --db standard_db
Bracken	Results derived from information produced by Kraken
MetaCache	> metacache-build-refseq
mOTUs	Marker database is pre-built and provided with software
MetaPhlAn	MetaPhlAn 2 was run using the marker dataset v296_CHOCOPhlAn_201901
